# Ibn-al-Nafis: An Ophthalmologist Who First Correctly Described the Circulatory System

**DOI:** 10.1167/tvst.10.2.17

**Published:** 2021-02-12

**Authors:** Shandiz Tehrani

**Affiliations:** 1 Casey Eye Institute, Oregon Health & Science University, Portland, OR, USA. e-mail: tehrani@ohsu.edu

I read with interest the *TVST* editorial by Dr. Van Gelder^1^ on physician-scientists in vision research. Kudos to Dr. Van Gelder for calling attention to this important subject matter.

Given my interest in the history of medicine, I had some background knowledge into many of the early clinician-scientists mentioned in the first paragraph of this editorial. Of note, although William Harvey is credited with elucidating the circulatory system, many medical historians would cite Ibn-al-Nafis, an Arab physician from the 1200s, who first challenged Galen's and Avicenna's centuries-old and incorrect assumptions about human circulation. Ibn-al-Nafis correctly mapped the pulmonary circulation and disproved the previously held dogma that blood passes through invisible holes between the right and left sides of the heart.^2^ And to top it all off, he was a prominent ophthalmologist by training.^3^

After personal correspondence with Dr. Van Gelder regarding the above information, he kindly suggested that I write to you “so others don't make this mistake.”
